# Isolation, Identification, and Determination of the Virulence of the Causal Agents of Corm Rot of Saffron (*Crocus sativus* L.) in Valle de Uco, Argentina

**DOI:** 10.3390/plants12142717

**Published:** 2023-07-21

**Authors:** Pablo F. Caligiore-Gei, Natalia Moratalla-López, Luciana M. Poggi, Gonzalo L. Alonso

**Affiliations:** 1 Estación Experimental Agropecuaria La Consulta, Instituto Nacional de Tecnología Agropecuaria, Ex Ruta 40 km 96.5, La Consulta, Mendoza 5567, Argentina; poggi.luciana@inta.gob.ar; 2 Cátedra de Química Agrícola, ETS de Ingeniería Agronómica y de Montes y Biotecnología de Albacete, Universidad de Castilla-La Mancha, Campus Universitario, 02071 Albacete, Spain; nataliamoratallalopez@gmail.com (N.M.-L.); gonzalo.alonso@uclm.es (G.L.A.)

**Keywords:** *Fusarium oxysporum* Schltdl., saffron, fungi, spice, phytosanitary status

## Abstract

Saffron (*Crocus sativus* L.) presents an attractive opportunity for diversifying production and adding value, particularly for small-scale growers and family-based agriculture. However, the agamic propagation of the crop through corms raises concerns regarding disease dispersion. During the summers of 2013 and 2015, symptoms of corm rot were observed in saffron crops in La Consulta, Valle de Uco, Argentina. These symptoms manifested in the form of wilting plants and red-coloured areas on the surface of the corms, in some cases affecting deeper regions. This study aimed to isolate and identify the causal agent responsible for saffron corm rot while also comparing the virulence of four strains isolated on saffron plants. Consistent isolation of *Fusarium* spp. colonies from affected corms confirmed its association with the disease. The obtained isolates were inoculated into healthy corms, and the reproduction of symptoms was confirmed, as well as subsequent pathogen re-isolation. Morphological and molecular characterisation of the strains was performed using rDNA gene sequencing. Furthermore, disease progression was assessed with fitting epidemiological models to empirical data, which served as estimators of fungal strain aggressiveness. The results conclusively identified *Fusarium oxysporum* Schltdl. as the causal agent of corm rot, and variations in virulence were observed among the strains on the host plant. After basic molecular and pathological studies, it is postulated that the fungal strains possibly belong to the forma specialis *gladioli*, but further studies are necessary to confirm that. The present study provides findings that highlight the importance of early detection and the preservation of pathogen-free fields to sustain saffron cultivation. These findings may constitute the initial step for future projects aimed at understanding the epidemiology of the disease better, determining the species/races of the pathogen, and developing effective management strategies.

## 1. Introduction

Saffron (*Crocus sativus* L.) is a monocotyledonous spice crop, believed to have originated in Persia. This spice has been cultivated since ancient times, and it is highly valued for its red stigmas. When dried, these stigmas, either with or without a yellow-white style portion attached, yield the spice, which possesses distinctive colouring, flavouring, and aromatic properties. The spice owes its colouring capacity, bitter taste, and pleasant aroma to its main compounds: crocetin esters, picrocrocin, and safranal. These compounds play a crucial role in defining the quality of saffron [1]. In addition to its culinary applications, saffron has been recognized for its medicinal properties throughout history. It is currently employed for its biomedical and pharmacological benefits. Evidence of saffron’s significance can be traced back to at least 1627 BC, as depicted in the frescoes discovered on the Greek island of Santorini. These frescoes showcase an offering of saffron stigmas to the goddess Thera. Remarkably, saffron cultivation has seen minimal technological advancements since then [1,2]. Currently, the global production of saffron is not well documented, but it is known that Iran accounts for 90% of the world’s production, with about 400 tons per year and more than 100,000 people employed [3]. In 2021, the top exporters were Iran (USD 104 million), Spain (USD 47 million), Afghanistan (USD 40.1 million), Greece (USD 10.4 million), and the United Arab Emirates (USD 7.47 million) [4]. The structure and conditions of the national and international saffron markets contrast with the characteristics found in the production areas, such as their limited size (except for Iran), strong local and traditional character, and an astonishing richness based on their traditional values, as saffron is a crop with hardly any mechanisation in which labour predominates. However, at present, there are several structural factors that are threatening the viability of these farms, such as the lack of family continuity, the shortage of labour, the effects of periodic rodent and rabbit damage, a shortage of adequately sized and healthy corms, climate change, international competition, and the serious difficulties encountered by small producers in gaining access to large commercial networks, in which it is essential to obtain high-quality saffron [5,6,7].

Saffron cultivation requires a specific climate, characterised by warm and dry summers and cold winters [8]. The biological and agronomic characteristics of *Crocus sativus* cultivation, such as reduced activity during summer, minimal fertilizer requirements, and adaptability to poor soil conditions, make this crop an attractive option for low-input agriculture and sustainable farming systems. It is estimated that saffron cultivation was initiated in Argentina during the 19th century through Spanish and Italian immigration [9]. Since then, it has successfully acclimatized to the region’s agroecology, although current production remains modest and primarily undertaken by small-scale family farming systems. The growing aspirations in saffron cultivation have prompted local producers to unite and consolidate their small-scale productions. Additionally, they are striving to develop distinct marketing channels that can enhance the value of their product. Over the past decade, Argentina has been importing approximately 3600 kg of saffron thread annually. However, achieving self-sufficiency in the national industry is currently impractical due to the limited and costly local production, along with the significant labour demands and phytosanitary aspects, which pose the primary challenges in scaling up production. Valle de Uco, located in the central-western region of Argentina, has optimal conditions for growing specialty crops, due to the suitable climate, particularly the ideal temperature variations, which enhances the essential oil content and quality of the spice. Consequently, saffron production presents an interesting alternative for family farming, to provide an additional activity to the production system [9].

Since saffron flowers are sterile, the plant does not produce viable seeds, and propagation is achieved vegetatively through the subterranean organs known as corms. Each season, new corms develop above the old ones, similarly to gladiolus corms. However, corms are susceptible to diseases caused by fungi, bacteria, nematodes, and viruses [10] and may constitute a way for disease dispersion. Hence, healthy starting corms are essential to ensure the sustainability of the crop and to prevent disease outbreaks [5]. One of the most destructive diseases that affect saffron is *Fusarium* corm rot. Infected plants in the field perish prematurely, leading to a reduction in corm yield, diminished quality, and decreased flower and stigma production [11]. This disease can also manifest during corm dormancy, whether in the field or during storage. The primary symptoms occur during the flowering period and are characterised by drooping, damping-off, yellowing, and wilting of shoots, as well as basal stem rot and corm rot. *Fusarium oxysporum* Schlechtend: Fr. f. sp. *gladioli* Massey (FOG) is the causal agent of this disease [8,12,13], which has been previously reported in Argentina [14], although no studies have been conducted since 1997. The pathogen survives in infected corms, plant debris, and the soil, in the form of mycelium, chlamydospores, macroconidia, and microconidia [12,15]. Infection can occur in the field when germinating spores or mycelium enter directly into the roots or through wounds. The pathogen can also be introduced into new saffron-growing regions through contaminated corms [8,16]. Currently, the disease is widespread, causing significant yield losses in various regions and a substantial decline in the cultivated areas across producer countries, primarily due to the consecutive cultivation and planting of infected corms [12]. Up to 50% plant mortality has been observed in severely affected fields [11]. Corm rot prevalence is a major limiting factor that impacts on saffron production [17] by hampering the multiplication of new corms and restricting the expansion of cultivation areas. Therefore, the greatest difficulty currently facing the saffron sector is the availability of a sufficient number of healthy starting corms.

**Figure 1:**
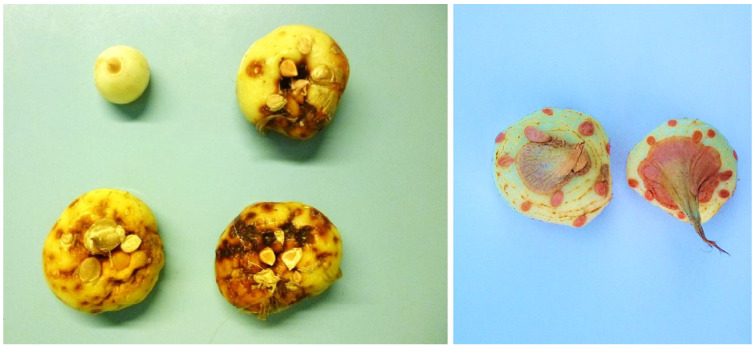
Symptoms of *Fusarium* corm rot in saffron, with different levels of severity (**left**); healthy asymptomatic corms (**right**).

During the harvest seasons of 2013 and 2015, we observed wilting and yellowing plants with decayed corms in the La Consulta region, situated in the heart of Valle de Uco, Argentina. The symptoms varied in severity, with some cases displaying a dry, deep reddish-coloured rot that affected the entire corm (see Figure 1). Consequently, the affected corms were deemed unsuitable for propagation and discarded. The purpose of this study was to identify the causal agent of the disease and investigate potential variations in the virulence of the isolates.

## 2. Results

### 2.1. Pathogen Isolation and Pathogenicity

Four isolates were obtained from the corms that exhibited symptoms. Subsequent pathogenicity tests confirmed that these isolates were indeed capable of inducing the same symptoms observed previously when inoculated onto healthy saffron corms. In these tests, corm rot manifested on both the surface and the deep tissues of the corms, sometimes affecting the entire structure. Conversely, the control corms, despite being mechanically injured by a cork borer, did not display any symptoms. This outcome validates the suitability of the described technique for assessing the pathogenicity of *Fusarium* in saffron corms in a rapid and effective manner. Consistently, *Fusarium*-like colonies were successfully re-isolated from the decaying tissues, fulfilling Koch’s postulates, confirming them as the causal agents of the disease.

### 2.2. Isolates Identification

Various morphological characteristics were observed and recorded. Microscopic examination of microcultures on SNA (Spezieller Nährstoffarmer agar) revealed the presence of oval, or slightly elliptical, unicellular microconidia arranged in false heads, as well as chlamydospores. The absence of polyphialids was also observed, consistent with the typical main features of the *Fusarium oxysporum* species. These observations provided further support for the identification and classification of the isolates [11,18]. Furthermore, the ribosomal DNA sequences obtained from the isolates were uploaded to the GenBank database (Table 1). These sequences were then compared to the sequences stored in the database. This comparative analysis enabled the identification of the isolates, based on their genetic similarities with known sequences [19]. The query sequences were in complete alignment with strains belonging to the *Fusarium oxysporum* Schltdl. species. Specifically, the isolates displayed 100% homology with four strains (DQ279794.1, DQ279795.1, MH854908.1, MH857680) identified as FOG (*Fusarium oxysporum* f. sp. *gladioli* [Massey] W.C. Snyder & H.N. Hansen).

### 2.3. Virulence Assay

**Figure 2:**
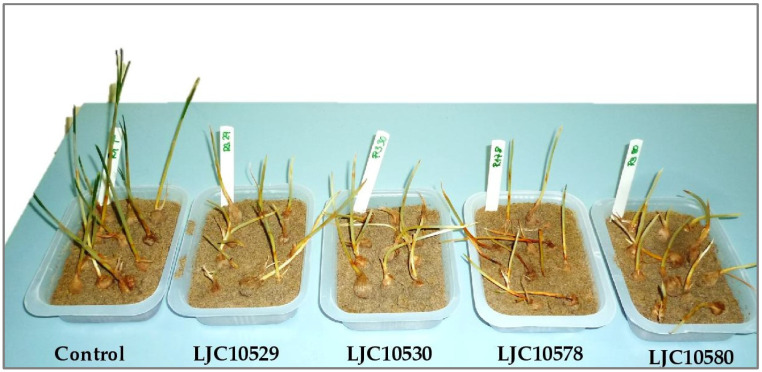
Virulence assay. Comparison of four *Fusarium* strains in controlled conditions. Symptoms of yellowing and wilting in the inoculated pots. Control pots show healthy plants.

The inoculated plants exhibited a range of symptoms consistent with *Fusarium* corm rot, including progressive yellowing and downward curling of the leaves, as well as rot in the basal plate and core of the corm. Shoot wilting, as depicted in Figure 2, was also observed, and it resembled the symptoms observed in the field closely. In contrast, the control plants remained healthy throughout the experiment and showed no signs of *Fusarium*-related symptoms.

**Figure 3:**
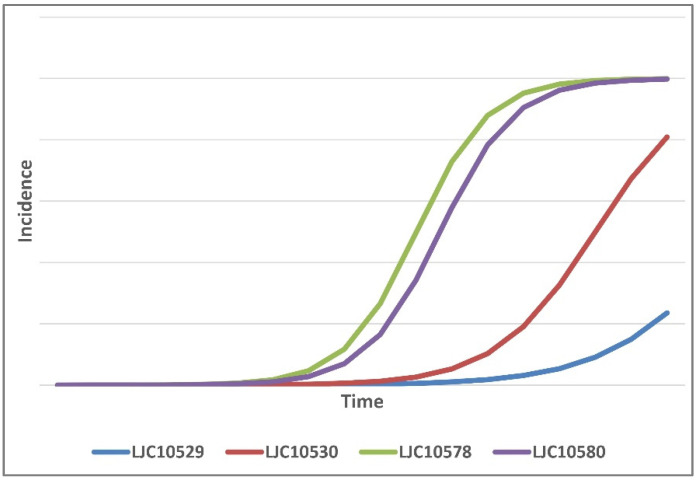
*Fusarium* corm rot disease progression over time across the four *Fusarium* strains, as modelled by linear regression (logistic model). Differences in virulence are suggested.

The incidence of diseased plants was analysed, which revealed highly significant effects of the treatment (i.e., *Fusarium* isolate) on the incidence variable (*p* < 0.01). Table 2 presents the results of the incidence of dead plants on days 19 and 21. The disease progression varied depending on the specific treatment, as illustrated in Figure 3. All isolates induced disease in the plants within a three-week period. Notably, strains LJC10578 and LJC10580 exhibited the highest levels of aggressiveness (Table 2) and differed significantly from the other strains, which indicated distinct variations in virulence among the *Fusarium* strains.

In the statistical analysis, the logistic model demonstrated a better fit than the monomolecular and Gompertz models, as evidenced by an evaluation of residual plots (data not shown). Although the determination values were not exceptionally high (Table 3), the logistic model was chosen as an initial approach to identify and distinguish the virulence groups. Consequently, the coefficients of the logistic model were employed to construct disease progression curves for each strain, as illustrated in Figure 3. The logistic integrated model represents the disease incidence *y* at a given time *t* as follows: y = 1/[1 + exp(−y0* + rL × t)]; rL =slope [20].

The analysis of the disease progression, as depicted in the figure, revealed three distinct virulence profiles among the evaluated *Fusarium* strains. Group A, comprising the LJC10578 and LJC10580 strains, exhibited the highest level of aggressiveness, consistent with the findings from the ANOVA analysis mentioned earlier. The disease progression curves for the Group A strains showed an earlier onset that increased over time compared to the other isolates (Figure 3), which was attributed to higher rates or slopes (as shown in Table 3). Group B, comprising LJC10530, demonstrated an intermediate level of virulence. Its disease progression curve displayed characteristics that were between the highly aggressive Group A and the less-virulent Group C (Figure 3). In contrast, Group C, encompassing LJC10528, exhibited the lowest level of virulence among the isolates tested. The disease progression curve for Group C showed a slower increase over time, compared to the other groups (Figure 3).

Overall, these observations suggest that there are distinct virulence categories among the evaluated *Fusarium* strains, with Group A being the most aggressive, Group B showing intermediate virulence, and Group C being the least virulent.

## 3. Discussion

Corm rot caused by *F. oxysporum* poses a significant threat to saffron crops worldwide, particularly in regions with suitable climatic conditions [8,11]. The long-term persistence of fungal inoculum in the soil exacerbates the problem, especially in perennial monocropping systems [12,15]; to mitigate this, rotation systems with a minimum of three years are recommended [9]. However, the asexual propagation of saffron plants, the ability of the pathogen to survive as a saprophyte, and the complex nature of soil-borne diseases such as *Fusarium* corm rot present additional challenges [17]. Currently, there are no effective management strategies available, such as resistant cultivars or fungicides, which makes the use of pathogen-free propagation corms the best approach for preventing the disease and maintaining pathogen-free fields [8,21].

The severity of the *F. oxysporum* strains in the soil may vary, and this needs to be validated by data [13]. Isolating and characterising the *Fusarium* strains responsible for corm rot is crucial to be able to assess their epidemiological importance and potential as a source of field outbreaks and seedborne inoculum [8]. Understanding the genetic diversity and virulence of different *Fusarium* strains is essential, not only to manage the disease effectively but to check if the obtained isolates correspond to pathogenic or saprophytic strains [22]. However, there is a lack of comprehensive studies on the causal agent of saffron corm rot, which emphasises the need for further research in this area. Examination of the genetic diversity of the pathogen and evaluation of the practical strategies to combat the disease are ongoing and can provide valuable insights for saffron growers. Although the major symptoms of the disease typically occur during the flowering period [8], the resting summer period, when corms remain in the soil and are dormant, may also contribute to pathogen infection due to more favourable climatic conditions, particularly optimal soil temperatures for *Fusarium* growth [12]. Recent studies have shown that latent inoculum of *F. oxysporum* can be found even in healthy corms [23].

Based on the modelled disease progression of the tested *Fusarium* strains, it was possible to hypothesise the presence of three virulence groups: Group A (LJC10578-LJC10580, more aggressive), Group B (LJC10530, intermediate), and Group C (LJC10528, less aggressive). The variation in virulence among the strains is in accordance with the findings in previous studies [13] and may have practical implications for saffron growers. The presence of highly virulent strains in propagation materials can pose a risk when introduced to pathogen-free areas, while the mildly aggressive strains may hinder early detection based on symptomatology. A comparison of the virulence of these groups with those evaluated in previous studies [8,11,13,21] could provide valuable insights into the composition of the *Fusarium* mycoflora associated with saffron corm rot in Valle de Uco, Argentina. In addition, fitting epidemiological models to empirical data can be useful for the comparison of isolates and the prediction of certain parameters, e.g., setting the optimal time for the evaluation of saffron accessions in screening tests for resistance to *Fusarium* corm rot.

The assay conducted in this study to test the virulence of the *Fusarium* strains followed a methodology that simulates the natural occurrence of the disease, prompting natural fungal infection instead of injuring the corms. Therefore, healthy corms were immersed in fungal suspensions and planted in inoculated substrate, leading to the development of symptoms typically associated with *F. oxysporum*, such as wilting and corm rot, all of which is consistent with previous research [11]. This inoculation method proved to be suitable for studying the virulence of *Fusarium* strains in this pathosystem and can be applied to investigations of other significant soilborne pathogens of saffron, such as *Penicillium* sp. or *Rhizoctonia* sp. [12,13,17].

Based on the results of the molecular studies, the evaluated strains could be identified as *Fusarium oxysporum* f.sp. *gladioli* (Massey) W.C. Snyder & H.N. Hansen (FOG). It is generally accepted that all *F. oxysporum* isolates that cause diseases in iridaceous crops, including saffron, belong to this forma specialis [11]. However, further molecular studies, including the sequencing of other genes, are necessary to confirm this identification. In addition, conducting cross-inoculation tests and further pathogenic studies can aid in assigning the strains to the correct forma specialis and even to specific races (virulence types) [8,11]. Given the presence of FOG in saffron-producing areas, it is advisable to avoid rotating these fields with ornamental crops such as gladiolus or narcissus, which are known to be hosts of the pathogen. However, the survival ability of the pathogen as a saprophyte [13] may reduce the effectiveness of crop rotation as a control measure. As a result, alternative approaches, including the use of biocontrol agents, are being considered. Our research group is currently testing native strains of *Trichoderma* spp. as biocontrol agents. These strains are applied as a treatment during planting to protect saffron corms from soil-borne *Fusarium* inoculum and directly target the inoculum on the corms. The assays show promising results for the management of the disease and could eventually be included in an integrated pest management programme.

The findings of this study may provide valuable insights for designing and improving strategies to manage *Fusarium* corm rot in saffron, particularly regarding early detection and the avoidance of the use of infected corms as propagules [12]. As saffron cultivation offers an attractive alternative for family farming [9], the preservation of pathogen-free fields is a significant concern, considering the various limitations, such as the unavailability of healthy corms, genetic erosion of the species, and difficulties for mechanisation [12]. The prevalence of arid soils in the Valle de Uco region may also contribute to the induction of infection, as suggested by some reports [12]. These findings are particularly relevant to local saffron growers, as no research has been conducted in this region, and the only record for the country was published decades ago [14]. Ongoing research is currently underway to investigate the aetiology of the disease, explore the genetic diversity of the pathogen, and evaluate management measures aimed at minimising the impact on saffron cultivation.

In conclusion, the findings highlight the importance of early detection, the avoidance of infected corms, and the preservation of pathogen-free fields to sustain saffron cultivation. Further research is needed to understand the genetic diversity and virulence of the pathogen and develop effective management strategies.

## 4. Materials and Methods

### 4.1. Pathogen Isolation

Rotten corms were detected during the 2013–2015 seasons. The corms showed reddish, dry, rotten tissues, both on the surface and in the inner parts. Differences in severity were detected. Pieces of approximately 10 corms from of each growing season were used for isolation purposes. Tissue was extracted from the advancing zone of the lesions and superficially disinfected by rinsing in 70% ethanol, 5% commercial bleach, and sterile distilled water (SDW). The disinfected pieces were air dried in a laminar flow chamber over sterile filter paper. They were then cut into small pieces (5 mm) and placed on the surface of Petri dishes containing potato dextrose agar (PDA). The dishes were incubated at 20 °C. After seven days, fungal colonies developed, and four *Fusarium*-like isolates were obtained from all of the samples.

### 4.2. Pathogenicity (Koch Postulates)

The pathogenicity of the isolates was checked through the inoculation of healthy saffron corms as follows. The corms were surface disinfected as stated above. A 4 mm cork borer was used to make holes in the equatorial zone, and 4 mm agar plugs taken from the edge of actively growing fungal colonies were inserted into the lesions. Control corms were inoculated with PDA plugs. Three replicates were performed for each isolate. The treated corms were incubated for one week. Rot only developed in the corms inoculated with the fungal colonies, and the symptoms corresponded with the original reddish dry rot. Re-isolation from these new lesions, performed as stated above, confirmed the pathogenicity of the isolates. The obtained strains were monosporised, incorporated into the Fungal Culture Collection of the Laboratorio José Crnko (WDCM 904, https://ccinfo.wdcm.org/details?regnum=904, accessed on 20 May 2023), and catalogued with the following accession numbers: LJC10529, LJC10530, LJC10578 and LJC 10580. The strains are available upon request.

### 4.3. Isolates Identification

The *Fusarium*-like isolates were grown on SNA (Spezieller Nährstoffarmer agar) [18] via the microculture technique [24]. The microscopical morphological features of each isolate were recorded, specifically the presence of chlamydospores and the shape of macroconidia and microconidia disposed in false heads. Genomic DNA was extracted from fungal mycelia for molecular identification purposes, employing the Wizard Genomic DNA Purification Kit (Promega Corp., Madison, WI, USA). The identity of the isolates was determined via polymerase chain reactions (PCR), using the ITS1/ITS4 primer pair. The PCR reaction mix contained buffer 1X, 0.025 U/µL of polymerase (GoTaq DNA polymerase, Promega Corp.), 12.5 µM of dNTPs, 0.30 µM of each primer, and 2 µL of DNA template, in a final volume of 50 µL. SDW served as a negative control. The reactions were carried out in a Bioneer MyGenie 96 Gradient Thermal Block thermocycler. The conditions set for the reaction were as follows: initial denaturation of two minutes at 94 °C, followed by 35 cycles of 60 s at 94 °C, 60 s at 53 °C, and 60 s at 72 °C, with a final elongation step of five minutes at 72 °C. The PCR products were checked by electrophoresis (agarose 2% in TBE buffer, 90 V, 105 min), using GelGreen (Biotium Inc., Fremont, CA, USA) as nucleic acid stain and visualised in a Dark Reader DR46B (Clare Chemical Research, Dolores, CO, USA) transilluminator. The PCR products were purified with the Bioneer Accuprep PCR Purification Kit (Bioneer Corp., Daedeok-gu, Daejeon, Republic of Korea) and sequenced by Macrogen Inc. (Geumcheon-gu, Seoul, Republic of Korea). The obtained sequences were edited and compared with the hosted sequences in GenBank [25].

### 4.4. Virulence Assay

Four *Fusarium oxysporum* isolates (LJC10529, LJC10530, LJC10578, and LJC 10580) were grown on potato dextrose agar plates for 10 days (28 °C). Micelia were collected, suspended in SDW, and filtered through sterile cotton filters. The obtained microconidial suspensions were quantified using a hemocytometer and counted under a microscope. The concentration was adjusted by adding SDW. The adjusted microconidial suspensions were used to inoculate pots that contained 300 g of sterile sand to a final concentration of 10,000 microconidia/g. Saffron corms (average size 1.6 g) were inoculated via immersion in fungal suspensions (concentration 1,000,000 microconidia/mL) for 60 min. Afterwards, the inoculated corms were air dried in a laminar flow chamber over sterile filter paper before being planted in the inoculated pots. Control treatments, with the use of SDW instead of fungal suspensions, were included. Twelve corms were planted in each pot, with three replicates for each isolate. The pots were randomly placed in a growing chamber (12 h light at 25 °C; 12 h darkness at 21 °C). Healthy plants were recorded in each pot at days 13, 17, 19, and 21 after inoculation.

To compare the virulence of the isolates, data were transformed to the incidence of dead plants (I = n/N) and evaluated on days 19 and 21 after inoculation, comparing the mean incidence among isolates through an analysis of variance (ANOVA). In addition, the incidence data were linearised, and linear regression analyses were performed to obtain the parameters of three classical epidemiological models: monomolecular, logistic, and Gompertz [20]. Residuals, graphic fit to experimental data, and R^2^ values were considered for model selection. The normal distribution of the data was also checked. The apparent infection rate (slope) parameter of the equation for every replicate was determined from linearised data. The slopes were then analysed by ANOVA and used for back-transformation to build a simulated disease progress curve (considered initial incidence y_0_ = 0.0001; y_0*_ = ln[(y_0_)]).

## Figures and Tables

**Table 1:** rDNA sequences ^1^ of the four evaluated *Fusarium* strains isolated from rotten corms and submitted to GenBank.

*Fusarium* Strain ^2^	GenBank Accession Number
LJC10529	OR028871
LJC10530	OR028872
LJC10578	OR028873
LJC10580	OR028874

^1^ The sequences correspond to the small subunit ribosomal RNA gene, partial sequence; internal transcribed spacer 1; 5.8S ribosomal RNA gene; internal transcribed spacer 2; large subunit ribosomal RNA gene, partial sequence. ^2^ LJC Laboratorio José Crnko (World Data Centre for Microorganisms #904).

**Table 2:** Virulence assay. Incidence of dead plants on days 19 and 21, among the four evaluated *Fusarium* strains. SD: standard deviation.

Treatment	Incidence Day 19	SD	Incidence Day 21	SD
LJC10580	0.583 a	0.220	0.778 a	0.048
LJC10578	0.583 a	0.300	0.778 a	0.255
LJC10530	0.111 b	0.127	0.667 ab	0.083
LJC10529	0.083 b	0.300	0.523 b	0.048
Control	0.000 b	0.000	0.028 c	0.048

Values with different letters differ significantly (95%) according to Duncan’s Test.

**Table 3:** Adjustment of epidemiological models to empirical data. Linear regression coefficients for each tested *Fusarium* strain and model.

Fusarium Strain	Model	Const	Slope	p-Value	R^2^
LJC10529	Logistic	−10.7437	0.2814	0.0437	0.2773
LJC10529	Gompertz	−2.6525	0.0781	0.0440	0.2767
LJC10529	Monomolecular	−0.1178	0.0206	0.0533	0.2578
LJC10530	Logistic	−10.8332	0.3611	0.0121	0.3951
LJC10530	Gompertz	−2.7230	0.1023	0.0165	0.3680
LJC10530	Monomolecular	−0.1733	0.0307	0.0520	0.2604
LJC10578	Logistic	−10.038	0.5012	0.0003	0.6794
LJC10578	Gompertz	−2.5446	0.1547	0.0015	0.5816
LJC10578	Monomolecular	−0.1570	0.0552	0.0251	0.3529
LJC10580	Logistic	−10.3444	0.4846	0.0007	0.5979
LJC10580	Gompertz	−2.7011	0.1556	0.0008	0.5924
LJC10580	Monomolecular	−0.2473	0.0580	0.0046	0.4723

## Data Availability

The data presented in this study are available on request from the corresponding author.
